# Analysis of the common complications and recurrence-related factors of superior parasagittal sinus meningioma

**DOI:** 10.3389/fsurg.2022.1023021

**Published:** 2023-01-06

**Authors:** Wei-Wei Chen, Yong Wang, Yang-Chun Hu, Yuan-Li Zhao

**Affiliations:** ^1^ Department of Neurosurgery, Beijing Tiantan Hospital, Capital Medical University, Beijing, China; ^2^ Department of Neurosurgery, The First Affiliated Hospital of Anhui Medical University, Hefei, China

**Keywords:** meningioma, complication, recurrence, superior parasagittal sinus, neurosurgery

## Abstract

**Objectives:**

Parasagittal meningioma resection is prone to postoperative complications and tumor recurrence because the tumor invades the superior sagittal sinus. This study aimed to clarify the incidence of perioperative complications and the recurrence of superior sagittal paranasal meningiomas and explored potential predictors in this context.

**Methods:**

The study retrospectively reviewed the clinical, imaging, and follow-up data of parasagittal meningiomas among patients who underwent microsurgical resection in the authors' institution from January 2008 to December 2017. Univariate and multivariate logistic regression analyses were conducted to explore independent predictors of perioperative complications and tumor recurrence.

**Results:**

A total of 212 parasagittal meningioma patients were included in this study. The incidence of perioperative complications was 23.6% (50/212), and perioperative death occurred in 6 (2.8%) patients. In univariate and multivariate logistic regression analyses of perioperative complications, peritumoral edema ≥1 cm (odds ratio [OR] 2.163, 95% confidence interval [CI] 1.057–4.428, *P* = 0.035) and the Sindou invasion Class V-VI(OR0.018, 95% CI 1.248–11.064, *P* = 0.018) were independent predictors. After an average of 83 (39–154) months of clinical follow up among the living 206 patients, 22 (10.7%) patients showed tumor recurrence. In univariate and multivariate logistic regression analyses of tumor recurrence, the Sindou invasion Class III-IV (OR 5.539, 95%CI 1.469–20.884, *P* = 0.011) and the Sindou invasion Class V-VI (OR 9.144, 95%CI 2.215–37.757, *P* = 0.002) were independent predictors.

**Conclusions:**

Peritumoral edema ≥1 cm and the Sindou invasion Class V-VI were the independent predictors of perioperative complications, and the Sindou invasion Class III-IV and the Sindou invasion Class V-VI were the independent predictors of tumor recurrence. The part of the parasagittal meningioma involving the sinus wall should be actively removed to the largest degree possible to reduce the recurrence rate.

## Introduction

The superior parasagittal sinus is one of the frequent locations of intracranial meningiomas, accounting for approximately 20%–30% of the total number (1, 2). The treatment of superior parasagittal meningiomas is challenging because these tend to invade the superior sagittal sinus (SSS) to varying degrees, resulting in stenosis or even occlusion of the sagittal sinus, which, in turn, affects venous return around the SSS involving important peripheral drainage veins. Total resection of extrasinus meningiomas can be achieved by microsurgery; however, the resection of intrasinus meningiomas is more challenging because it can sometimes be difficult to obtain a good separation interface as a result of intraoperative bleeding.

In the past, scholars have proposed radical resection of meningiomas in the sagittal sinus and even used venous bypass to replace the affected sagittal sinus to reduce the tumor recurrence rate (3, 4). However, this procedure is relatively complex, and, furthermore, the affected sagittal sinus is sometimes accompanied by thick drainage veins. Therefore, an increasing number of researchers do not advocate for the radical replacement of the SSS. Concurrently, with the continuous development of stereotactic radiosurgery, an increasing number of scholars believe that residual tumors in the sinus could nonetheless be treated using this method to obtain a good tumor progression-free survival rate, which can effectively reduce complications (5, 6). However, some researchers believe that the occurrence of procedural complications involving superior parasagittal meningioma is not closely related to the presence of a residual meningioma in the sinus. Sometimes, even if the meningioma in the sinus is not treated, it can still aggravate brain edema and venous infarction (7). The complications of radiosurgery, such as a sudden increase in volume of hemorrhagic residual tumor, edema aggravation, and scalp necrosis cannot be ignored (8, 9).

Due to a lack of large samples and long-term clinical data, it is difficult to understand the complications and recurrence-related factors of parasagittal meningiomas. This study summarized the clinical data of 212 patients with parasagittal meningiomas who underwent surgery in the authors' department (single center) from January 2008 to December 2017. The complications and recurrence-related factors of parasagittal meningioma were collected and statistically analyzed.

## Materials and methods

### Subjects

This study's protocol conformed to the ethical guidelines of the 1975 Declaration of Helsinki and was approved by the Institutional Ethics Review Committee of the First Affiliated Hospital of Anhui Medical University; informed consent was obtained from all patients to confirm their inclusion in the research (ethics no., 2019-11-08).

The clinical data of patients with parasagittal meningiomas diagnosed by imaging and pathology in the authors' hospital from January 2008 to December 2017, were retrospectively analyzed. The enrolled patients were required to have complete clinical data, including imaging examinations, hospitalization records, and follow-up outpatient records (including telephone follow-up data); patients operated on initially for recurrent parasagittal meningioma were excluded. All of the enrolled patients were slated to receive telephone or email follow-up replies before December 1, 2021.

### Examinations

All the patients were examined by magnetic resonance imaging (MRI) and MR venography (MRV)/digital subtraction angiography before surgery. The Sindou classification of parasagittal meningiomas was determined using the MRI examination. The maximum axial distance from the meningiomas to the peripheral edema was used to evaluate the peritumoral edema according to the T2 MRI series signal before surgery. The Karnofsky performance status (KPS) was used to evaluate the neurological function of patients before surgery, at discharge, and six months after surgery. The degree of surgical resection was evaluated according to the surgery record and MRI examination six months after the operation. The surgical complications included postoperative intracerebral hematomas, aggravation of brain edema, epilepsy, as well as medical complications (pulmonary embolism, venous thrombosis, and disturbance in water and electrolyte balances), as well as any new neurological deficit or the aggravation of previous neurological deficit symptoms during the perioperative period.

### Surgeries

The surgeries were performed by the senior neurosurgery staff of the authors' institution according to the standard microsurgical procedures. All the meningiomas outside of the SSS were removed using the conventional method (intratumor decompression combined with tumor wall separation), and attention was paid to protecting the surrounding drainage veins. According to the preoperative Sindou classification, for Sindou types I and II parasagittal meningiomas (the tumors of which involved the side-wall of the sagittal sinus and did not affect blood flow in the sinus), radical resection of the tumor was performed, including tumor fragments in the crypt of the sagittal sinus. Electrocoagulation of the lateral wall of the sagittal sinus was performed, and the sinus wall was repaired using a gelatin sponge, muscle, or fascia to achieve gross total resection (GTR). For Sindou types III and IV sagittal sinus meningiomas (where the tumor involved most of the side wall of the sagittal sinus and affected, but did not block, blood flow in the sinus), the side wall that had been invaded by the SSS was opened as far as possible, and the intrasinus part of the tumor was removed. The residual lateral wall of the SSS was then sutured with fascia, a patch, or a gelatin sponge to obtain the largest degree of gross tumor resection possible. However, in cases where there were large drainage veins on the lateral wall (particularly when the tumor was located in the parietal lobe), the surgeon was more cautious about opening the lateral wall of the sagittal sinus.

The authors achieved subtotal resection (STR) for Sindou types V and VI parasagittal meningiomas, i.e., a tumor involving the entire lateral wall of the sagittal sinus and blocking blood flow of the sagittal sinus. Here, the meningioma in the sinus was removed along the natural gap between the tumor and the dura mater. If the meningioma was located in the front third of the sagittal sinus, the latter could be ligated. Special attention was given to protect the common compensations of peripheral venous return, such as the inferior sagittal sinus. Otherwise, the upper sagittal sinus wall was opened, and as much of the tumor in the sinus wall as possible was removed, except for tightly adhered parts and sections with a tough texture.

### Evaluation

Simpson grade I refers to complete tumor resection with removal of affected dura and bone, while grade II refers to complete tumor resection with coagulation of affected dura. Simpson grade III refers to complete tumor resection. Simpson grade IV refers to the subtotal tumor resection, with a small amount of tumor residue visible on a postoperative MRI scan. Simpson grade V refers to the decompression with/without biopsy. Simpson grades I, II, and III resections are classified as GTR, while grades IV and V resection are characterized as STR.

For patients with grade II/III meningiomas [according to World Health Organization (WHO, 2007) classification], regardless of the degree of resection, conventional radiotherapy was used in the region where surgery had been conducted following the procedure. All patients underwent routine MRI enhancement and MRV examination six months after surgery; thereafter, an MRI examination was performed once a year. Stereotactic radiosurgery was recommended if the MRI showed small residual lesions six months following the surgery. The recurrence of parasagittal meningiomas was defined as the presence of a new enhanced mass in GTR cases or a significant increase in the residual mass in STR cases six months after surgery.

### Statistical analysis

The SPSS 22.0 was used for statistical analysis in this study. The chi-squared test was used for univariate analysis, and a logistic regression analysis was conducted for multivariate analysis. Fisher's exact test was used in less than five cases. The KPS score was measured using a rank-sum (Mann–Whitney U or Wilcoxon) test. A *P*-value less than 0.05 was considered statistically significant.

## Results

### Baseline characteristics

From January 2008 to December 2017, 264 patients with parasagittal meningiomas, diagnosed by imaging and pathology, underwent their first surgical treatment in the neurosurgery department of the authors' hospital. Finally, 212 patients were enrolled in the current study. The remaining 52 patients were not included due to follow-up loss or incomplete case data. The participants included 143 females (67.4%) and 69 males (32.6%). The average patient age at the time of surgery was 54.3 years (25–80 years). Among the patients, 111 (52.3%) had parasagittal meningiomas that were located in the middle third of the sagittal sinus, 66 (31.1%) had parasagittal meningiomas located in the anterior third of the sagittal sinus, and 35 (16.6%) had tumors located in the posterior third of the sagittal sinus. A total of 147 patients (69.3%) had tumors larger than 4 cm in diameter. A total of 122 patients (57.5%) who had parasagittal meningiomas had peritumoral edema that encompassed a diameter larger than 1 cm (10, 11). A total of 161 patients (75.9%) who had parasagittal meningiomas received GTR, and 69 patients (32.5%) who had parasagittal meningiomas received STR. A total of 186 (87.7%) superior parasagittal meningiomas were classified as WHO grade I tumors, 23 (10.9%) superior parasagittal meningiomas were denoted as WHO grade II tumors, and 3 (1.4%) parasagittal meningiomas were recorded as being WHO grade III tumors (see Table 1).

**Table 1:** Features and outcomes of 212 patients with superior parasagittal meningiomas.

Parameter	N (%)
Sex	
Female	143 (67.4%)
Male	69 (32.6%)
Median age at surgery in yrs (range)	54.3 ± 11.23 (25–80)
Involved superior sagittal sinus 3rd	
Anterior 1/3	66 (31.1%)
Middle 1/3	111 (52.3%)
Posterior 1/3	35 (16.6%)
The largest diameter at surgery in cm	
<4 cm	65 (30.6%)
≥4 cm	147 (69.3%)
Grade of resection	
Simpson	
I	50 (23.6%)
II	91 (42.9%)
III	20 (9.4%)
IV	51 (24.1%)
V	0 (0.0%)
WHO grade	
I	186 (87.7%)
II	23 (10.9%)
III	3 (1.4%)
Sindou grade	
I-II	157 (74.1%)
III-IV	39 (18.4%)
V-VI	16 (7.5%)
Peritumoral edema	
<1 cm	90 (42.5%)
≥1 cm	122 (57.5%)
Preop.KPS	
≥90	126 (59.4%)
≤80	86 (40.6%)

### Risk factors for complications

There were 50 cases (23.6%) of perioperative complications in 212 patients with superior parasagittal meningiomas (see Table 2 for details). Among them, 25 (11.8%) had venous infarction/cerebral edema, 14 (6.6%) had intracerebral hematoma, and seven (3.3%) had new onset epilepsy. There were four patients (1.9%) with perioperative drug complications, such as pneumonia, urinary tract infection, incision infection, and deep vein thrombosis. Six patients (2.8%) died postoperatively, three (1.4%) died perioperatively, and two patients (0.9%) died of cerebral herniation caused by postoperative intracerebral hematoma. Although the hematomas were surgically removed, the prognosis of the patients remained poor. The patient (0.5%) with postoperative cerebral edema died of postoperative cerebral edema, and the prognosis was poor following decompressive craniectomy. Two patients (0.9%) had malignant tumors and died of malignant tumor progression three months after discharge. One patient (0.5%) died of chronic obstructive pulmonary disease complicated with pulmonary heart disease and delayed pulmonary infection after discharge (see Table 2.).

**Table 2:** The complications among 212 patients with parasagittal meningiomas.

Complications	No (%)
Venous infarct/cerebral edema	25 (11.8%)
Brain hematoma	14 (6.6%)
Medical complication	4 (1.9%)
New seizures	7 (3.3%)

Univariate analysis was conducted to analyze the possible factors related to perioperative complications, and the possible risk factors included age, sex, the KPS score on admission, the maximum diameter of the tumor, the location of the sagittal sinus, peritumoral edema, the Sindou classification of tumor invasion of the sagittal sinus, the grade of resection, and the postoperative WHO pathological results. The peritumoral edema, the largest diameter and the Sindou invasion showed statistical significance for the occurrence of perioperative complications (*P* < 0.05). The multivariate analysis showed that the peritumoral edema and the tumor invasion of the sagittal sinus were independent risk factors for perioperative complications (*P* < 0.05). The risk of complications in patients with Sindou types V-VI was significantly higher than those in patients with Sindou types I-II (OR = 3.716, 95%CI: 1.248–11.064, see Table 3).

**Table 3:** Predictive factors of 212 patients with parasagittal meningiomas who had complications based on univariate and multivariate analysis.

Variables	No (%)	Univariate		Multivariate	
		*P* value	OR	95%CI	*P* value
Sex					
Male	20/69 (28.9%)	0.198	ND		
Female	30/143 (20.9%)				
Age					
≥70	4/16 (25.0%)	0.548	ND		
<70	46/196 (23.5%)				
Location					
Anterior 1/3 SSS	19/66 (28.8%)	0.263	ND		
Middle 1/3 SSS	26/111 (23.4%)				
Posterior 1/3 SSS	5/35 (14.3%)				
Largest diameter					
<4 cm	8/65 (12.3%)	0.010	2.303	0.988–5.366	0.053
≥4 cm	42/147 (28.6)				
Peritumor Edema					
<1 cm	14/90 (15.6%)	0.018	2.163	1.057–4.428	0.035
≥1 cm	36/122 (29.5%)				
Sinus invasion					0.042
Class I-II	30/157 (19.1%)	0.011	1.00		
Class III-IV	12/39 (30.8%)		1.726	0.770–3.873	0.185
Class V-VI	8/16 (50.0%)		3.716	1.248–11.064	0.018
Grade of resection					
Simpson		0.083	ND		
I	13/50 (26.0%)				
II	16/91 (17.6%)				
III	3/20 (15.0%)				
IV	18/51 (35.3%)				
Pathology					
I	42/186 (22.6%)	0.212	ND		
II	6/23 (26.1%)				
III	2/3 (66.7%)				
Preop.KPS					
≥90	25/126 (19.8%)	0.120	ND		
≤80	25/86 (29.1%)				

### Risk factors for recurrence

The survival of 206 patients with superior parasagittal meningiomas was evaluated during the follow-up period. Up to December 2020, the average follow-up time was 83 (39–154) months. A total of 22 patients were found to have tumor recurrence. The recurrence rate of Simpson grade I resection was 6.1% (3/49), grade II resection recurrence was 7.8% (7/90), grade III resection recurrence was 10.5% (2/19), and grade IV resection recurrence was 20.8% (10/48).

A univariate analysis was conducted to analyze the possible factors related to recurrences, such as age, sex, the KPS score on admission, the KPS score at discharge, the KPS score six months after surgery, the tumor size, location of the sagittal sinus, peritumoral edema, the Sindou classification of sagittal sinus invasion, the resection grade, and the WHO pathological results. The grade of resection, the Sindou classification of sagittal sinus invasion, adjuvant radiotherapy and WHO pathological classification were statistically significant for tumor recurrence (*P* < 0.05). The multivariate analysis showed that the Sindou classification of sagittal sinus invasion and the WHO pathological classification were independent risk factors for tumor recurrence (*P* < 0.05). The risk of recurrence in patients with Sindou types III-IV (OR = 5.539, 95%CI: 1.469–20.884, see Table 4) and types V-VI (OR = 9.144, 95%CI: 2.215–37.757, see Table 4) were significantly higher than those in patients with Sindou types I-II.

**Table 4:** Predictive factors of 206 patients with parasagittal meningiomas having recurrence based on the univariate and multivariate analyses.

Variables	No (%)	Univariate		Multivariate	
		*P* value	OR	95%CI	*P* value
Sex					
Male	9/65 (13.8%)	0.318	ND		
Female	13/141 (9.2%)				
Age					
≥70	2/15 (13.3%)	0.730	ND		
<70	20/191 (10.5%)				
Location					
Anterior 1/3 SSS	4/64 (6.3%)	0.382	ND		
Middle 1/3 SSS	14/109 (12.8%)				
Posterior 1/3 SSS	4/33 (12.1)				
Largest diameter					
<4 cm	3/65 (4.6%)	0.056	ND		
≥4 cm	19/141 (13.5%)				
Peritumor Edema					
<1 cm	8/87 (9.2%)	0.555	ND		
≥1 cm	14/119 (11.8%)				
Sinus invasion					0.004
Class I-II	7/153 (4.6%)	0.000	1.00		
Class III-IV	10/38 (26.3%)		5.539	1.469–20.884	0.011
Class V-VI	5/15 (33.3%)		9.144	2.215–37.757	0.002
Grade of resection					
GTR (Simpson I,II and III)	12/158 (7.6%)	0.007	0.919	0.221–3.819	0.907
STR (Simpson IV)	10/48 (20.8%)				
Pathology					
I	16/181 (8.8%)	0.021	2.192	0.554–8.674	0.263
II-III	6/25 (24.0%)				
Adjuvant Radiotherapy					
Yes	13/59 (22.0%)	0.001	0.783	0.183–3.344	0.741
No	9/147 (6.1%)				

### The recovery of neurological function after surgery

The preoperative KPS score of 126 patients (59.5%) was above 90, and 10 patients (4.7%) had a KPS score below 70. At discharge, 121 patients (57.0%) had KPS scores above 90, while 27 (12.6%) had KPS scores below 70, including three patients who died during the perioperative period. The KPS score of 187 patients (90.7%) was above 90, and only four (1.9%) had residual lifelong neurological dysfunction. Overall, there was no significant difference in the KPS scores comparing preoperative and discharge status (*P* > 0.05), but the KPS score was significantly improved after six months (*P* < 0.05) (Table 5).

**Table 5:** The KPS evaluation at the preoperation, discharge and late time periods.

KPS	Preoperative Evaluation (%)	Discharge Evaluation (%)	Late Evaluation (%)
100	12 (5.7%)	9 (4.2%)	93 (45.1%)
90	114 (53.8%)	112 (52.8%)	94 (45.6%)
80	76 (35.8%)	64 (30.2%)	15 (7.3%)
≤70	10 (4.7%)	27 (12.6%)^a^	4 (2.0%)^b^
Total	212	212	206

^a^ Three patients died in the perioperative period, and 2 patients died of postoperative intracerebral hematoma which caused a cerebral hernia, Although the hematoma was removed surgically, the patient still died, One patient died of sudden brain swelling after the operation, and the effect of decompressive craniectomy was poor.

^b^ Three patients died after discharge. Two patient were complicated with a malignant tumor. Three months after discharge, these patients died due to the progression of the original malignant tumor. One patient died due to chronic obstructive pulmonary disease complicated with pulmonary heart disease and persistent pulmonary infection.

## Discussion

Surgery for parasagittal meningioma remains challenging and controversial. According to the literature statistics, only 25% of patients with superior parasagittal meningiomas can clinically obtain a Simpson grade I resection (9). Many researchers have proposed replacing the SSS with a vein graft and a vascular bypass after resecting the tumor and the attached SSS wall to achieve recanalization of the SSS and Simpson grade I resection of the tumor, which can reduce the recurrence rate (3, 4, 12–14). However, this manipulation prolongs the surgery time and increases the difficulty and incidence of postoperative complications. There were no significant differences between Simpson grades I–III tumors for the recurrence of SSS meningiomas (2, 15, 16). In the current study group, intrasinus meningiomas were removed to the largest possible degree to reduce recurrence, and the affected SSS was repaired with materials such as fat, meningeal patches, and fascia rather than a vein graft or vascular bypass.

The current literature reports the incidence of postoperative parasagittal meningioma complications as 15%–30% (5, 9, 15, 17). The incidence of complications in the current group was 23.6%. The incidence of postoperative cerebral hematoma, postoperative venous infarction, and aggravation of cerebral edema was much higher, and the multivariate analysis showed that peritumoral edema and the Sindou grade of tumor invasion of the SSS were independent risk factors for perioperative complications (*P* < 0.05). For superior parasagittal meningiomas, 57.5% of patients had peritumoral edema with a maximum diameter larger than 1 cm. Venous drainage of the tumor may change the original peripheral venous drainage method, increasing the pressure of local venous reflux of the SSS. If the peripheral cortical venous vessels are fully compensated, peritumoral edema may not be obvious; however, if venous compensation is insufficient, the peripheral cortical venous return will be blocked, which may lead to severe peritumoral edema, and tumor tissue often closely adhering to the surrounding cortex. During surgery, cortical damage can easily be caused due to fragile peripheral edema, which can increase the incidence of postoperative cerebral hematoma, SSS vein infarction, and brain edema (18–21).

There are many classification methods for meningiomas of the SSS. The Sindou classification is widely used (3) and was also applied to the data of the authors' group. For meningiomas classified at a Sindou grade higher than III, the tumor broadly involved the lateral wall of the sagittal sinus, and the meningioma volume in the sinus was relatively large. Accordingly, the tumor will have a degree of impact on the venous reflux of the sagittal sinus, and the incidence of surgical complications will be relatively higher. Collateral circulation may exist more widely in tumors with a higher Sindou grade for superior parasagittal meningiomas; therefore, patients with such tumors are prone to postoperative complications, e.g., aggravated brain swelling due to the insufficient establishment of collateral circulation (1, 22). The present study found the Sindou grade to be an independent risk factor for perioperative complications involving superior parasagittal meningiomas (*P* < 0.05). Over time, little permanent dysfunction will remain following the establishment of collateral circulation.

Because it grows beside the sagittal sinus, it has abundant blood supply. So the recurrence rate of parasagittal meningiomas is relatively high (23, 24). Sindou (3) reported that 100 cases of parasagittal meningiomas were completely removed with radical resection; the recurrence rate was 4%, and the mortality rate was 3%, with follow up for an average of eight years. However, this low recurrence rate was judged according to postoperative clinical manifestations. There was no imaging evidence, and, as such, a degree of bias may have been present in the results. Colli et al. (25) reported that in 53 patients with parasagittal meningiomas, 45 underwent total resection, seven underwent subtotal resection, and one patient underwent partial resection. After 7.8 years of follow up, 17 patients had recurrence (recurrence rate, 32.7%), which included a WHO grade III recurrence rate of 100%, WHO grade II recurrence rate of 75%, and WHO grade I recurrence rate of 17.5%.

In recent years, with the continuous development of stereotactic radiosurgery technology, an increasing number of studies have suggested that subtotal resection of the intrasinus tumor of superior parasagittal meningiomas, supplemented by postoperative stereotactic radiosurgery and other multimodal methods, could achieve good tumor progression-free survival rates (5, 26–28). Mathiesen et al. (26) reported that, among 21 patients with superior parasagittal meningiomas treated by subtotal resection for various reasons, the tumor progression rate was 73%, while the progression rate after gamma knife treatment was 10%. Patients who regarded gamma knife treatment as part of the initial approach during the early stage could obtain a 90% tumor control rate. Patients treated with gamma knife treatment after tumor progression only obtained a 50% tumor control rate. The study stressed that the gamma knife method should not be regarded as a first choice for the treatment of parasagittal meningiomas but should be used as adjuvant treatment after surgery to control tumor growth. After analyzing 114 patients, Mair et al. (28) reported that adjuvant radiotherapy could only obtain significant benefits when patients had a subtotal resection but found that radiotherapy had no significant benefits for patients who had a total resection (Simpson resection degree I–II). Hardesty et al. (29) found that radiotherapy after total tumor resection did not affect the progression-free survival rate. Therefore, close follow-up observation can be used as a reasonable treatment strategy for patients with total tumor resection. The data in the present group advocated that parasagittal meningiomas (except those involving the central vein in the parietal lobe) should be removed to the largest extent possible. For residual sinus meningiomas following surgery, stereotactic radiosurgery should be actively carried out instead of observation. After tumor progression, stereotactic radiosurgery should be added to the treatment protocol. The data of the present group were followed up for an average of 95 months, and the recurrence rate was 10.7%. Compared with other similar literature reports, these results were satisfactory.

Some studies have shown that parasagittal meningiomas are prone to clinical or imaging recurrence three to four years after surgery (30–32). The recurrence time of anaplastic and malignant parasagittal meningiomas is shorter; the average recurrence times of the latter were 13 months in the present group. Patients with postoperative pathological grade WHO II and III meningiomas, regardless of the degree of resection, were routinely treated with ordinary radiotherapy in the area where surgery had been performed. In addition, statistical analysis of the data showed that the Sindou grade of meningiomas involving the sagittal sinus was an independent risk factor for postoperative meningioma recurrence (*P* < 0.05). This may be related to the wide range of sagittal sinus involvement of a Sindou V–VI meningioma, which may even include the surrounding venous complex.

Total resection of meningioma in the sinus, which was conducted for the patients in the present study, was sometimes difficult. It has been reported that tumor cells also exist 4 cm around the tumor mass, visible to the naked eye (33). Moreover, researchers also found that the recurrence of parasagittal meningiomas was not always entirely in the exposed surgery area but was also found near the cutting edge and could involve a wide range of tumors. During surgery, particularly in the parietal lobe, insufficient exposure to parasagittal meningiomas can lead to the omission, growth, and recurrence of tumor cells, and this requires urgent research attention.

The limitation of the current study is that the included cases were all patients who completed the follow-up protocols and had good compliance. In cases where patients were lost to follow up, there may be parasagittal meningioma recurrence; these patients may have given up on treatment or may have been transferred to other hospitals. Accordingly, patients who are lost to follow up may impact the final results of the research to some extent. Moreover, the lack of family wise error correction in this study is also one of the limitations.

## Conclusion

In this study, peritumoral edema and the specific Sindou grade were independent risk factors for postoperative complications of parasagittal meningiomas. The Sindou grade was independent risk factors for the recurrence of parasagittal meningiomas. The risks of complications and recurrence were significantly higher in patients with Sindou grade III-IV or V-VI than those with Sindou grade I-II. The part of parasagittal meningiomas that involves the sinus wall should be actively resected to the largest extent possible to reduce the recurrence rate. For malignant parasagittal meningiomas, it is necessary to use general or stereotactic radiosurgery for residual tumor sections in addition to surgical resection.

## Data Availability

The original contributions presented in the study are included in the article/Supplementary Material, further inquiries can be directed to the corresponding author/s.

## References

[1] Della Puppa A Rustemi O Gioffrè G Rolma G Grandis M Munari M Application of indocyanine green video angiography in parasagittal meningioma surgery. Neurosurg Focus. (2014) 36(2):E13. 10.3171/2013.12.FOCUS13385 24484251

[2] Munich SA Eddelman D Byrne RW. Retrospective review of a venous sparing approach to resection of parasagittal meningiomas. J Clin Neurosci. (2019) 64:194–200. 10.1016/j.jocn.2019.02.013 30876934

[3] Sindou MP Alvernia JE. Results of attempted radical tumor removal and venous repair in 100 consecutive meningiomas involving the major dural sinuses. J Neurosurg. (2006) 105(4):514–25. 10.3171/jns.2006.105.4.514 17044551

[4] Bederson JB Eisenberg MB. Resection and replacement of the superior sagittal sinus for treatment of a parasagittal meningioma: technical case report. Neurosurgery. (1995) 37(5):1015–9. 10.1227/00006123-199511000-00026 8559326

[5] Gatterbauer B Gevsek S Höftberger R Lütgendorf-Caucig C Ertl A Mallouhi A Multimodal treatment of parasagittal meningiomas: a single-center experience. J Neurosurg. (2017) 127(6):1249–56. 10.3171/2016.9.JNS161859 28156245

[6] Mathiesen T Pettersson-Segerlind J Kihlström L Ulfarsson E. Meningiomas engaging major venous sinuses. World Neurosurg. (2014) 81(1):116–24. 10.1016/j.wneu.2013.01.095 23376533

[7] Sindou M. Meningiomas involving major dural sinuses: should we attempt at radical removal and venous repair? World Neurosurg. (2014) 81(1):46–7. 10.1016/j.wneu.2013.07.119 23924964

[8] Buerki RA Horbinski CM Kruser T Horowitz PM James CD Lukas RV. An overview of meningiomas. Future Oncol. (2018) 14(21):2161–77. 10.2217/fon-2018-0006 30084265 PMC6123887

[9] Sheehan JP Cohen-Inbar O Ruangkanchanasetr R Bulent Omay S Hess J Chiang V Post-radiosurgical edema associated with parasagittal and parafalcine meningiomas: a multicenter study. J Neurooncol. (2015) 125(2):317–24. 10.1007/s11060-015-1911-1 26329323

[10] Schneider M Güresir Á Borger V Hamed M Rácz A Vatter H Preoperative tumor-associated epilepsy in patients with supratentorial meningioma: factors influencing seizure outcome after meningioma surgery. J Neurosurg. (2019) 10(11):1–7. 10.3171/2019.7.JNS19455 31604333

[11] Della Puppa A Rustemi O Gioffrè G Rolma G Grandis M Munari M Application of indocyanine green video angiography in parasagittal meningioma surgery. Neurosurg Focus. (2014) 36(2):E13. 10.3171/2013.12.FOCUS13385 24484251

[12] Hakuba A Huh CW Tsujikawa S Nishimura S. Total removal of a parasagittal meningioma of the posterior third of the sagittal sinus and its repair by autogenous vein graft. Case report. J Neurosurg. (1979) 51(3):379–82. 10.3171/jns.1979.51.3.0379 469583

[13] Sindou M Mazoyer JF Fischer G Pialat J Fourcade C. Experimental bypass for sagittal sinus repair. Preliminary report. J Neurosurg. (1976) 44(3):325–30. 10.3171/jns.1976.44.3.0325 1249611

[14] Steiger HJ Reulen HJ Huber P Boll J. Radical resection of superior sagittal sinus meningioma with venous interposition graft and reimplantation of the rolandic veins. Case Report. Acta Neurochir (Wien). (1989) 100(3-4):108–11. 10.1007/BF01403595 2589116

[15] DiMeco F Li KW Casali C Ciceri E Giombini S Filippini G Meningiomas invading the superior sagittal sinus: surgical experience in 108 cases. Neurosurgery. (2004) 55(6):1263–74. 10.1227/01.neu.0000143373.74160.f2 15574208

[16] Ricci A Di Vitantonio H De Paulis D Del Maestro M Gallieni M Dechcordi SR Parasagittal meningiomas: our surgical experience and the reconstruction technique of the superior sagittal sinus. Surg Neurol Int. (2017) 8:1. 10.4103/2152-7806.198728 28217380 PMC5288983

[17] Gousias K Schramm J Simon M. The Simpson grading revisited: aggressive surgery and its place in modern meningioma management. J Neurosurg. (2016) 125(3):551–60. 10.3171/2015.9.JNS15754 26824369

[18] Jang WY Jung S Jung TY Moon KS Kim IY. Predictive factors related to symptomatic venous infarction after meningioma surgery. Br J Neurosurg. (2012) 26(5):705–9. 10.3109/02688697.2012.690914 22702388

[19] Magill ST Theodosopoulos PV McDermott MW. Resection of falx and parasagittal meningioma: complication avoidance. J Neurooncol. (2016) 130(2):253–62. 10.1007/s11060-016-2283-x 27778211

[20] Han MS Kim YJ Moon KS Lee KH Yang JI Kang WD Lessons from surgical outcome for intracranial meningioma involving major venous sinus. Medicine (Baltimore). (2016) 95(35):e4705. 10.1097/MD.0000000000004705 27583904 PMC5008588

[21] Yin T Zhang J Zhang H Zhao Q Wei L Wang S. Poor brain-tumor interface-related edema generation and cerebral venous decompensation in parasagittal meningiomas. World Neurosurg. (2018) 115:e544–51. 10.1016/j.wneu.2018.04.092 29689390

[22] Zeeshan Q Patel A Cheng CY Zhao NH Barber J Ghodke BV Resection of meningiomas involving Major dural venous sinuses: classification, technique, and long-term results. World Neurosurg. (2019) 125:e521–36. 10.1016/j.wneu.2019.01.128 30716491

[23] Nowak A Marchel A. Surgical treatment of parasagittal and falx meningiomas. Neurol Neurochir Pol. (2007) 41:306–14. 17874338

[24] Sagaonkar PS Pattanshetty R. Effect of medical qigong therapy on distress, fatigue, and quality of life in head and neck cancer patients undergoing intensity-modulated radiation therapy: a single arm clinical trial. World J Tradit Chin Med. (2021) 7:427–35. 10.4103/wjtcm.wjtcm_15_21

[25] Colli BO Carlotti CG Jr Assirati JA Jr Dos Santos MB Neder L Dos Santos AC. Parasagittal meningiomas: follow-up review. Surg Neurol. (2006) 66(Suppl 3):S20–8. 10.1016/j.surneu.2006.08.023 17081848

[26] Mathiesen T. Parasagittal meningiomas. Handb Clin Neurol. (2020) 170:93–100. 10.1016/B978-0-12-822198-3.00031-8 32586512

[27] Hadelsberg U Nissim U Cohen ZR Spiegelmann R. LINAC Radiosurgery in the management of parasagittal meningiomas. Stereotact Funct Neurosurg. (2015) 93(1):10–6. 10.1159/000368440 25501917

[28] Mair R Morris K Scott I Carroll TA. Radiotherapy for atypical meningiomas. J Neurosurg. (2011) 115(4):811–9. 10.3171/2011.5.JNS11112 21699480

[29] Hardesty DA Wolf AB Brachman DG McBride HL Youssef E Nakaji P The impact of adjuvant stereotactic radiosurgery on atypical meningioma recurrence following aggressive microsurgical resection. J Neurosurg. (2013) 119(2):475–81. 10.3171/2012.12.JNS12414 23394332

[30] Nowak A Dziedzic T Krych P Czernicki T Kunert P Marchel A. Benign versus atypical meningiomas: risk factors predicting recurrence. Neurol Neurochir Pol. (2015) 49(1):1–10. 10.1016/j.pjnns.2014.11.003 25666766

[31] Katz TS Amdur RJ Yachnis AT Mendenhall WM Morris CG. Pushing the limits of radiotherapy for atypical and malignant meningioma. Am J Clin Oncol. (2005) 28(1):70–4. 10.1097/01.coc.0000139958.88481.5c 15685038

[32] Conti A Pontoriero A Salamone I Siragusa C Midili F La Torre D Protecting venous structures during radiosurgery for parasagittal meningiomas. Neurosurg Focus. (2009) 7(5):E11. 10.3171/2009.8.FOCUS09-157 19877789

[33] Borovich B Doron Y. Recurrence of intracranial meningiomas: the role played by regional multicentricity. J Neurosurg. (1986) 64(1):58–63. 10.3171/jns.1986.64.1.0058 3941351

